# Characteristics of the effects of *Polygonati Rhizoma* on gut microbiota and metabolites in vitro associated with poor dietary habits in pregnant women

**DOI:** 10.1371/journal.pone.0314335

**Published:** 2024-12-05

**Authors:** Zhiwei Xu, Jiabin Li, Lue Hong, Yangli Zhang, Chunyu Wang, Hailong Yang, Lisha Zhao, Ping Qiu, Zhi Du, Hui Wang

**Affiliations:** 1 Jinhua Academy, Zhejiang Chinese Medical University, Jinhua, China; 2 Second Clinical Medical School, Zhejiang Chinese Medical University, Hangzhou, China; 3 Department of Pharmacy, Children’s Hospital, Zhejiang University School of Medicine, National Clinical Research Center for Child Health, Hangzhou, Zhejiang, China; 4 First Clinical Medical School, Zhejiang Chinese Medical University, Hangzhou, China; 5 School of Pharmaceutical Sciences, Zhejiang Chinese Medical University, Hangzhou, China; 6 Analysis and Testing Center, Zhejiang Academy of Traditional Chinese Medicine, Hangzhou, China; State Islamic University Sunan Kalijaga: Universitas Islam Negeri Sunan Kalijaga, INDONESIA

## Abstract

Poor dietary habits have been associated with dysbiosis and microbial imbalance in pregnant women. Such imbalances can pose health risks during pregnancy. This study aimed to explore the impact of *Polygonati Rhizoma* on the gut microbiota of pregnant women through *In vitro* simulated fermentation. Interestingly, significant differences in microbial community richness and structure were found between the control and the treatment with *Polygonati Rhizoma*. Analysis of composition and variability indicated that the treatment with *Polygonati Rhizoma* group showed higher levels of *Lactobacillus* and *Bifidobacterium*, but lower levels of *Parabacteroides* and *Lachnoclostridium*. The study also investigated specific genera differences between groups using the co-occurrence network analysis and their correlations with microbial metabolites by the redundancy analysis (RDA), Mantel-test network heatmap, and heatmap highlighting the relationships among gut microbiota, short-chain fatty acids (SCFAs), and gases in the absence or presence of Polygonati Rhizoma supplementation. Functional predictions from BugBase phenotype prediction indicated changes in potentially pathogenic and aerobic bacteria in Polygonati Rhizoma supplementation. Overall, the findings provide valuable insights into the influence of Polygonati Rhizoma on the gut microbiota in pregnant women associated with poor dietary habits.

## Introduction

Maternal dietary habits have been shown to significantly influence the composition and function of the gut microbiota during pregnancy [1]. Diets high in processed foods, sugars, and saturated fats, and low in fiber-rich fruits, vegetables, and whole grains, can lead to dysbiosis and microbial imbalances in expectant mothers [2]. Conversely, abalanced and diverse diet rich in prebiotic fibers, vitamins, and minerals has been linked to a healthier gut microbiota profile during pregnancy [3]. Additionally, intake of probiotic-rich and fermented foods, as well as dietary supplements, may positively influence the gut’s microbial composition and function, potentially lowering the risk of gut microbiota-related disorders in pregnant women [4,5]. However, further research is needed to elucidate the specific dietary components and mechanisms underlying this relationship and to develop targeted dietary interventions to enhance gut health in pregnant women.

Gut microbiological disorders during pregnancy can significantly affect both maternal health and fetal development. The maternal gut microbiota is essential for regulating immune responses, nutrient absorption, and hormonal balance, impacting the health of pregnant women [6–8]. Disruptions in microbial balance are linked to an increased risk of complications like gestational diabetes [9,10]. Moreover, changes in the maternal gut microbiota can lead to adverse outcomes for the child, such as a higher risk of neurodevelopmental disorders and compromised immune system development [11–13]. Addressing these disorders is crucial not only for maternal well-being but also for the optimal growth and development of the fetus. Although dietary modifications, probiotics, and antibiotics show promise in restoring microbial balance, their effectiveness varies, and there are significant gaps in understanding the most effective strategies [14]. Due to ethical constraints, many therapies effective for gut microbiota disorders have not been thoroughly studied in pregnant women, often leading to a perception of limited treatment options for these conditions during pregnancy [15]. This underscores the urgent need to develop a reliable *in vitro* method to systematically and credibly evaluate therapeutic approaches for managing microbiota disorders in pregnancy.

*Polygonati Rhizoma*, a traditional Chinese medicinal herb, has attracted attention for its potential impact on the gut microbiota. A number of studies have indicated that extracts of *Polygonati Rhizoma* may have prebiotic qualities, promoting the growth of beneficial bacteria such as *Bifidobacteria* and *Lactobacilli*, while inhibiting harmful pathogens. These beneficial effects are thought to be driven by bioactive components like polysaccharides and oligosaccharides, which serve as food sources for favorable gut bacteria [16–18]. Moreover, *Polygonati Rhizoma* extracts have demonstrated the ability to regulate the production of short-chain fatty acids (SCFAs), essential for maintaining gut health and immune function [19]. Additionally, *Polygonati Rhizoma* might also possess anti-inflammatory properties that enhance its positive influence on the composition and function of the gut microbiota [20]. However, further investigation is needed to delineate the precise mechanisms through which *Polygonati Rhizoma* affects the gut microbiota and its potential role in treating gut-related disorders during pregnancy.

To overcome ethical research barriers and concerns about fetal development, this study employs *In vitro* simulated fermentation to examine the impact of *Polygonati Rhizoma* on the gut microbiota and related metabolites in pregnant women with poor dietary habits. The research focuses on the modulation of microbiota and changes in the production of SCFAs and gases. The results of this study will contribute to the confirmation of *Polygonati Rhizoma* as a beneficial dietary supplement for pregnant women with poor dietary habits.

## Materials and methods

### Materials and reagents

*Polygonati Rhizoma* was procured from Zhejiang Tiantai Kun Huang Biotechnology Co.,Ltd., China. Bile salt, yeast extract, L-cysteine, and heme were obtained from Sigma Company, USA. Phosphate-buffered saline (PBS), NaCl, KH_2_PO_4_, K_2_HPO_4_, MgSO_4_, CaCl_2_, metaphosphoric acid, and crotonic acid were purchased from Sangon Biotech (Shanghai) Co., Ltd, China. The Yeast Extract-Casitone-Fatty Acids (YCFA) medium was procured from Dingguochangsheng Biotechnology (Beijing) Co., Ltd, China.

### Collection of fresh fecal samples from participants

The study included 10 pregnant women, aged between 22 and 35 years, at 37–41 weeks gestation, from March 20, 2024, to April 23, 2024. The study included participants who had a pre-pregnancy body mass index (BMI) in the normal range (18.5–24.9 kg/m^2^). All the participants underwent a 75-g oral glucose tolerance test (OGTT) between 24 and 28 weeks of pregnancy. GDM was diagnosed when OGTT value fulfilled at least one of the following criteria: FPG < 92 mg/dL, 1-h plasma glucose < 180 mg/dL, and 2-h plasma glucose < 153 mg/dL. The exclusion criteria were as follows: (1) smoking or alcohol consumption during pregnancy; (2) use of antibiotics or probiotics within the last month; and (3) presence of inflammatory bowel disease, infectious disease, cardiac, hepatic, or renal disease, immune disorders, psychiatric conditions, or malignancy. All participants were residents of Hangzhou, Zhejiang Province. This research was approved by the Ethics Review Committee of Sir Run Run Shaw Medical College Affiliated Hospital of Zhejiang University (No. 20240186), and written informed consent was obtained from all participants, and written informed consent was obtained from all participants. Fresh stool samples from the participants were collected and stored at 4°C, and processed within four hours.

### Quality of the dietary habits

The dietary habits of pregnant women were measured with the validated index of diet quality (IDQ) questionnaire [21]. This validated questionnaire evaluates a range of dietary components, including the consumption of fruits, vegetables, whole grains, proteins, and fats, as well as the intake of added sugars and sodium. Each participant receives a score that reflects the overall quality of their diet, with higher scores indicating better adherence to recommended dietary guidelines. A score below 10 points out of a possible 15 points was categorized as poor dietary habits and was included in our study. This validated questionnaire reveals inadequate consumption of essential food groups, such as fruits, vegetables, and whole grains, while often reflecting excessive intake of processed foods high in sugars and unhealthy fats.

### Treatment of fresh fecal samples

After obtaining informed consent from the participants or their parents, sterile fecal sample boxes (30 mL, 91 mm × Φ24 mm) provided by BioRexin Ltd (Shanghai, China) were used to collect the samples. A minimum of 3 grams of intermediate stool, characterized by low food residue and minimal air exposure post-defecation, was promptly selected and clearly labeled with the participant’s information. The collected samples were stored at 4°C and processed within 4 hours. The stool samples were then diluted in a ten-fold volume of PBS inside a fume hood and filtered to remove large particles using a 55 mm × 28 mm 300-mesh fecal sieve processor manufactured by Hua Tuo Co. (Shanghai, China) [22].

### *In vitro* simulated fermentation of gut microbiota

To prepare YCFA medium, the following were dissolved in water: 4.5 g/L yeast extract, 3.0 g/L tryptone, 3.0 g/L peptone, 0.4 g/L bile salts, 0.8 g/L cysteine hydrochloride, 4.5 g/L NaCl, 2.5 g/L KCl, 0.45 g/L MgCl_2_, 0.2 g/L CaCl_2_, and 0.4 g/L KH_2_PO_4_. Additionally, 1.0 mL of Tween 80, 1.0 mL of resazurin, and 2.0 mL of trace element solution were added. The mixture was then boiled and nitrogen was vented through it to maintain anaerobic conditions in the liquid medium. 4.5 mL of the medium was dispensed into 10 mL vials using a peristaltic pump manufactured by Longer Co., Ltd (Baoding, China). The vials were then sealed and autoclaved at 115°C and 101 kPa for 15 min using a heated pressure steam sterilizer manufactured by Shen An Co., Ltd. (Shanghai, China) [23]. The huangjing (HJ) group was treated with the power of *Polygonati Rhizoma* (0.1 g/5 ml of YCFA medium), while the control (CK) group with no additions to their YCFA medium.

### Genomic DNA extraction and 16S rRNA gene sequencing

After simulating fermentation *in vitro*, the diversity of the gut microbiota was analyzed via 16S rRNA sequencing. Genomic DNA from the microbial community was extracted using the FastDNA® Spin Kit for Soil (MP Biomedicals, USA), following the provided manufacturer’s instructions. The bacterial 16S rRNA was amplified using a thermocycling polymerase chain reaction (PCR) system (GeneAmp 9700, ABI, San Diego, CA, USA) with primer pairs 341F (5’-CCTAYGGGRBGCASCAG-3’) and 806R (5’-GGACTACHVGGTWTCTAAT-3’). The PCR conditions included a 3-minute pre-denaturation, 27 cycles of 95°C for 30 seconds each, annealing at 55°C for 30 seconds, extension at 72°C for 45 seconds, and a final extension at 72°C for 10 seconds. The resulting amplicons were then purified and sequenced from end to end using the NovaSeq PE250 platform (MajorbioBioPharm Technology Co. Ltd., Illumina, San Diego, USA) based in Shanghai, China, adhering to industry standards.

After quality control splicing, the raw sequences were denoised using the DADA2 plug-in within QIIME2 (version 2020.2), which produced amplicon sequence variants (ASVs). These ASVs were then categorized using the SILVA 16S rRNA database (v138) and the Naive Bayes classifier of QIIME2. α-diversity metrics, including Ace, Chao, and Sobs indices, were calculated based on the specified depths. β-diversity was assessed using the q2-diversity plug-in in QIIME2, where Bray-Curtis distances were computed.

### Measurement of SCFAs in simulated fermentation *in vitro*

After fermentation, the samples were subjected to centrifugation to separate the supernatant. This supernatant was then acidified using a mix of crotonic acid and metaphosphoric acid for 24 hours. Following acidification, it was centrifuged again and passed through a 0.22 μm aqueous microporous membrane filter (Millipore Express, Germany). Subsequently, 150 μL of the filtered supernatant was injected into a sample vial. The aging of the samples was conducted using a gas chromatograph (GC-2010 Plus, Shimadzu, Japan). The column temperature was increased from 80°C to 190°C at a rate of 10°C/min, then to 240°C at a rate of 40°C/min and held for 5 minutes. The flame ionization detector (FID) and the gasification chamber were both set to 240°C. The carrier gases used were nitrogen (20 mL/min), hydrogen (40 mL/min), and air (400 mL/min). The SCFAs in the culture filtrate were quantified using a DB-FFAP thin-film capillary column (30 m × 0.32 mm × 0.50 μm, Agilent Technologies, Inc., USA), with trans-2-butenoic acid used as the internal standard. The SCFAs identified were acetic acid (AA), propionic acid (PA), isobutyric acid (IBA), butyric acid (BA), isovaleric acid (IVA), and valeric acid (VA) [24].

### Measurement of gas in simulated fermentation *in vitro*

After 24 hours of fermentation, the gas production of the bottles was measured at room temperature (25°C) using a fermentation gas analyzer (Empaer, China) equipped with five sensitive gas sensors, as previously described [25]. Before starting measurements, the gas detector was activated and allowed to warm up. The inlet and outlet ports of the analyzer were connected to the syringe bottle using rubber tubing and a disposable syringe needle, ensuring the needle did not contact the liquid surface of the culture medium to avoid water contamination. The concentrations of the five gases, including CH_4_, H_2_S, NH_3_, CO_2_, and H_2_, were recorded at their peak values. Subsequent tests were conducted only when the concentration values of all five gases dropped to zero.

### Data analysis

The data was analyzed for statistical significance using SPSS 23.0 (IBM Corporation, USA) and expressed as mean ± standard error of the mean (SEM). The visual representations of the various gases, SCFAs, and five bacterial genera were generated using GraphPad Prism 8.0.1 (GraphPad Software, USA). For data following a normal distribution, paired t-tests were employed, while paired Wilcoxon rank sum tests were used for data that did not follow a normal distribution. Principal Coordinate Analysis (PCoA) was applied to illustrate the data using the Bray-Curtis distance matrix. Correlation heat maps were generated using Spearman’s correlation coefficient. Functional predictions were evaluated with BugBase phenotype prediction. All data analysis and plotting were performed on the Majorbio cloud platform (https://www.majorbio.com).

## Results

### ASVs analysis

**Fig 1:**
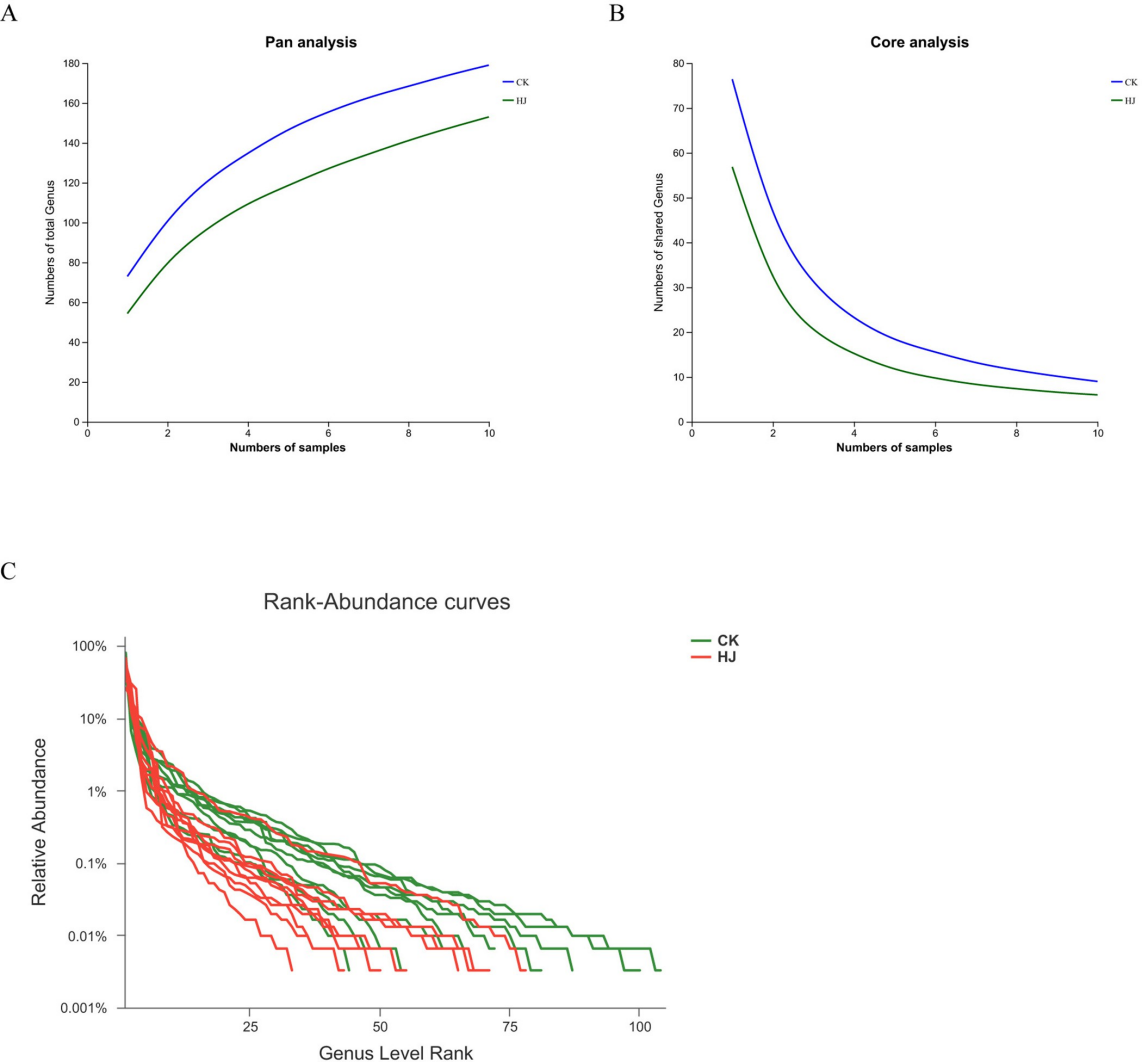
ASVs analysis of gut microbiota after *In vitro* simulated fermentation. (A) Pan analysis and (B) Core analysis between the CK and HJ groups. (C) Rank-abundance curves between the CK and HJ groups.

Fig 1A and 1B show the outcomes of the Pan genera and Core genera analyses, respectively. The former illustrates the total genera found across the samples, while the latter displays the shared genera to all samples. The investigation revealed that the trend in total genera did not exhibit a gradual leveling in any of the study groups. However, the trend of shared genera counts did demonstrate a gradual leveling off across all study groups, which suggests that the sample sizes are sufficient for determining the core genera count. Fig 4C indicates a wide and gradually narrowing spread on the horizontal axis, reflecting high genera richness and a uniform distribution of gut microbiota across the groups.

### Diversity analysis

**Fig 2:**
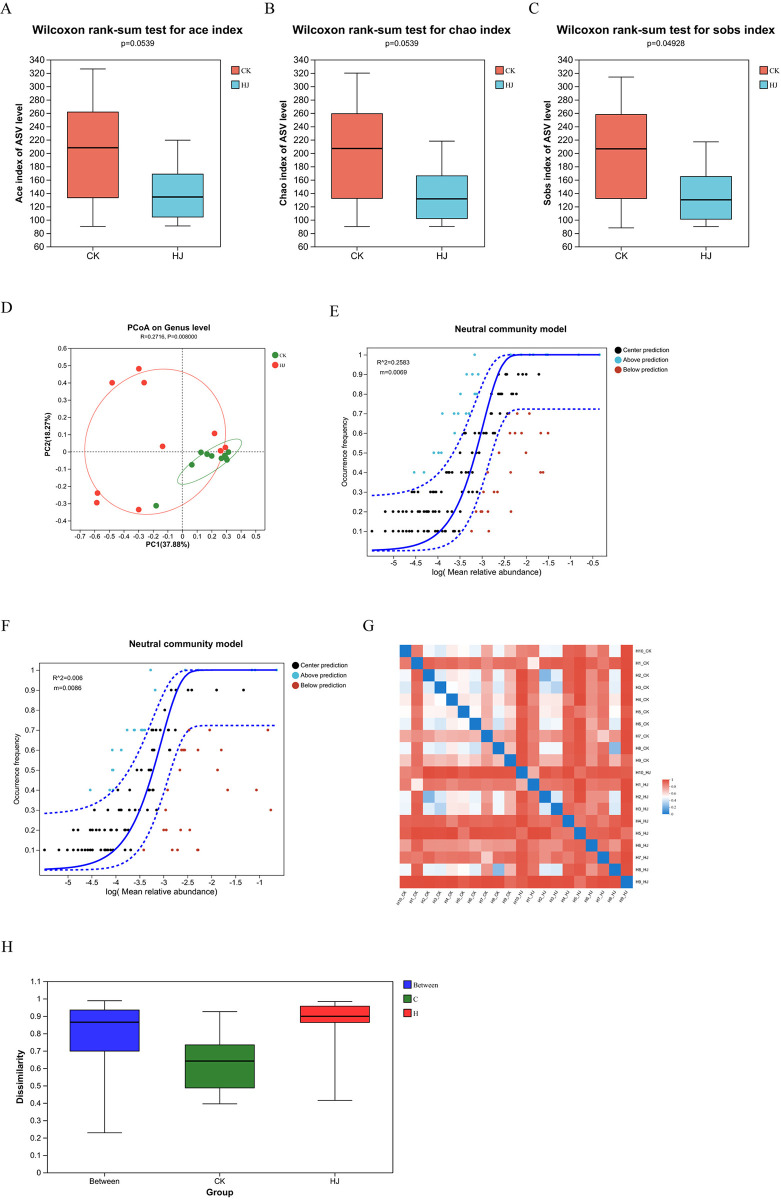
Diversity analysis of gut microbiota after *In vitro* simulated fermentation. (A-C) the α-diversity indices (Ace, Chao, and Sobs) comparison between the CK and HJ groups. (D) PCoA analysis between the CK and HJ groups. (E-F) Neutral community model analysis between the CK and HJ groups. (G) Sample distance matrix between the CK and HJ groups. (H) Inter-group distance box plot between the CK and HJ groups.

After *In vitro* simulated fermentation, we analyzed different gut microbiota using 16S rRNA sequencing. Fig 2A–2C displays the α-diversity of the CK and HJ groups. No statistically significant differences (P > 0.05) were observed in the Chao and Ace indices between the CK and HJ groups. However, the Sobs index indicated a statistically significant difference (P < 0.05) between the groups. Fig 2D–2H displays the β-diversity of the CK and HJ groups. The PCoA analysis (P = 0.008) demonstrated significant differences in the genus-level structure of the bacterial communities between the CK and HJ groups during *In vitro* simulated fermentation (Fig 2D). The neutral community model effectively described the relationship between the frequency of population occurrence and its relative abundance (Fig 2E and 2F). The R^2^ value for the HJ group (R^2^ = 0.0086) was higher than that for the CK group (R^2^ = 0.0069), suggesting that stochastic processes play a larger role in the intestinal biotope due to the HJ intervention. Hierarchical cluster analysis was conducted to illustrate differences in community distributions across environmental samples, based on the beta diversity distance matrix (Fig 2G and 2H). The results show that the distances between the CK and HJ groups were larger than those within each group, indicating significant disparities between the two microbial genera groups.

### Composition and difference analysis

**Fig 3:**
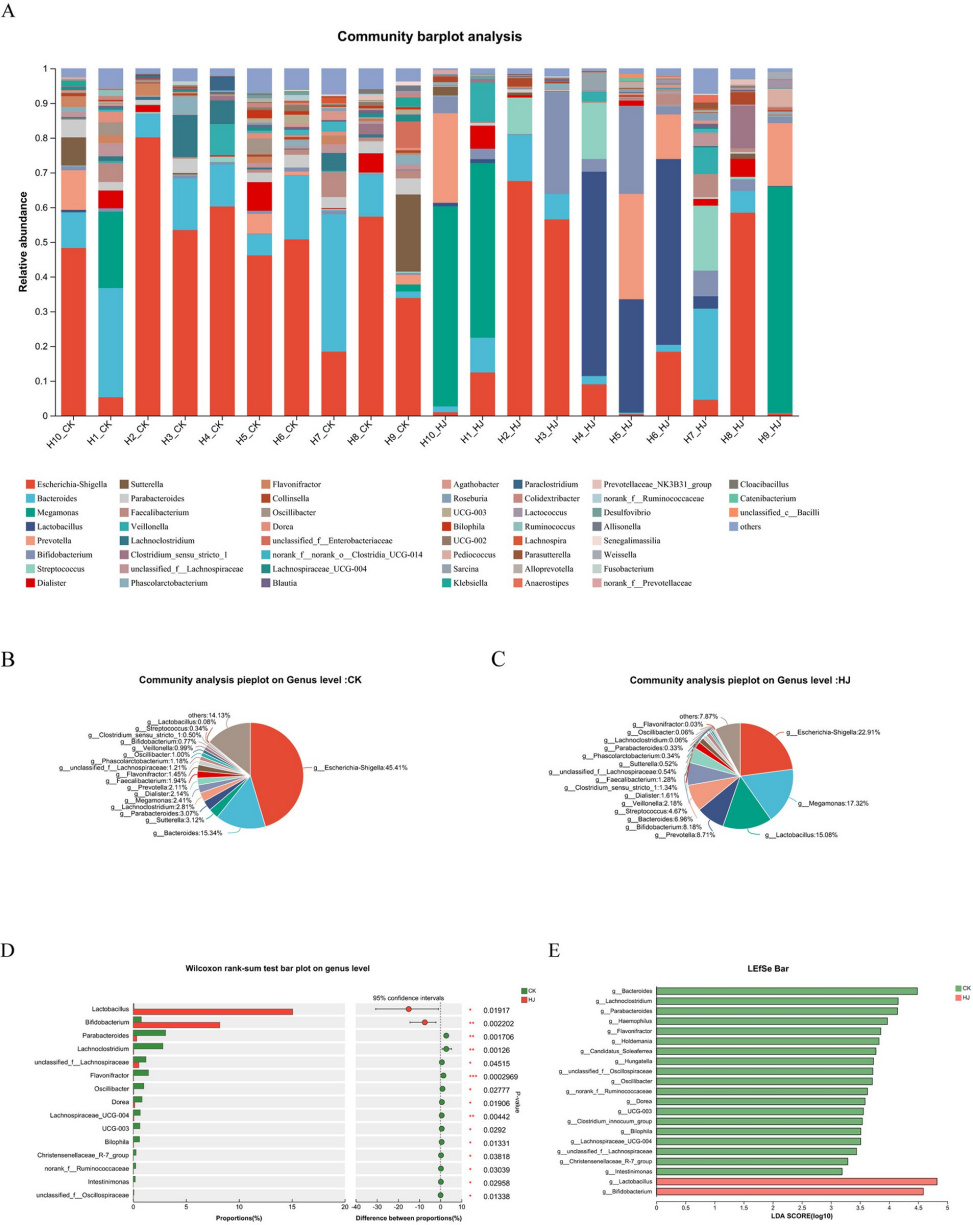
Analysis of gut microbiota composition and difference following *In vitro* simulated fermentation. (A) Community barplot analysis between the CK and HJ groups. (B-C) Pieplot analysis on genus level for HJ and CK groups. (D) Wilcoxon rank-sum test barplot on genus level between the CK and HJ groups. The significance thresholds for P-values are * 0.01 < P ≤ 0.05, ** 0.001 < P ≤ 0.01, *** P ≤ 0.001. (E) LEfSe barplot analysis between the CK and HJ groups on genus level (LDA > 2).

Fig 3A shows a bar graph of the relative abundance of different genera at the genus level in 20 fecal samples. In the CK group, *Escherichia-Shigella* was the most prevalent genus, constituting 45.41% of the microbiota, followed by *Bacteroides* at 15.34%, *Sutterella* at 3.12%, and *Parabacteroides* at 3.47% (Fig 3B). In the HJ group, *Escherichia-Shigella* also led with a relative abundance of 22.91%, followed by *Prevotella* at 15.34%, *Bifidobacterium* at 8.18%, and *Lactobacillus* at 6.96% (Fig 3C). Differences in specific bacterial taxa enrichment between the HJ and CK groups were highlighted through a between-group difference test and LEfSe analysis (Fig 3D and 3E). The Wilcoxon rank-sum test was employed to analyze differences in genera abundance between the two groups, based on community abundance data. This test aided to identify significant differences and potential microbial biomarkers for the CK and HJ groups, aiding in the development of predictive models. From the results of Fig 3D, it can be found that the relative abundance of *Lactobacillus* and *Bifidobacterium* was significantly increased in the HJ group compared to the CK group, while the relative abundance of *Lactobacillus*, *Clostridium*, *unclassified_f_Lachnospiraceae*, and *Flavonifractor* were significantly decreased. Fig 3E demonstrates that there was a significant difference in the abundance of 21 genera between the CK and HJ groups (LDA > 2), with *Lactobacillus* and *Bifidobacterium* significantly enriched in the HJ group, while *Bacteroides*, *Lachnoclostridium*, *Parabacteroides*, and *Heamophilus* were more prevalent in the fecal samples from the CK group.

### Relevance analysis

**Fig 4:**
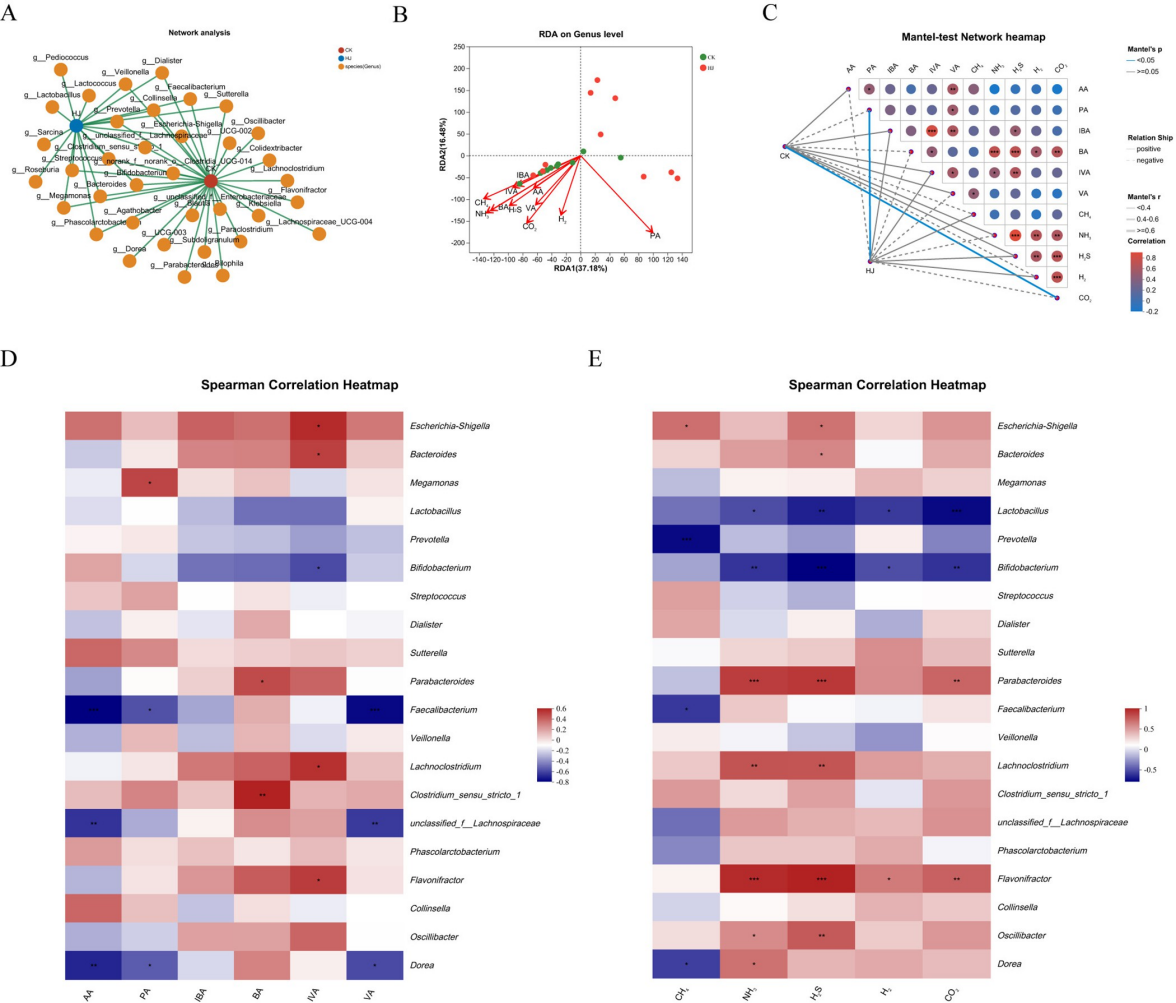
Relevance analysis of gut microbiota and metabolites after *In vitro* simulated fermentation. (A) The co-occurrence network between the CK and HJ groups. (B) Mantel-test network heamap between the CK and HJ groups. Line thickness reflects the strength of the correlation, measured by Mantel’s ’r’ (absolute value). Positive and negative correlations between the community and metabolites are shown, with different colors indicating the nature of the correlation and color intensity indicating the strength. (C) The RDA on genus level between the CK and HJ groups. (D-E) Spearman correlation heatmap of SCFAs and gases. The significance thresholds for P-values are * 0.01 < P ≤ 0.05, ** 0.001 < P ≤ 0.01, and *** P ≤ 0.001.

The co-occurrence network analysis results indicated that 17 genera were unique to the CK group and 5 were unique to the HJ group, with 16 genera shared between them (Fig 4A). The RDA scatterplot revealed that CO₂ was more pronounced in the CK group, whereas PA was more noticeable in the HJ group. (Fig 4B). The Mantel-test network heatmap revealed a significant and strong positive correlation between IBA and IVA, as well as between H_2_S and NH_3_ (Fig 4C). H_2_S and NH_3_ display a significant and strong positive correlations (P < 0.05, corr > 0.5) with *Parabacteroides*, *Lachnoclostridium*, and *Flavonifractor*, while exhibiting a significant and strong negative correlations (P < 0.05, corr < -0.5) with *Lactobacillus* and *Bifidobacterium* (Fig 4D). Additionally, CO_2_ exhibits significant and strong negative correlations (P < 0.05, corr < -0.5) with *Lactobacillus* and *Bifidobacterium*. Fig 4E indicates that PA is significantly and strongly correlated (P < 0.05, corr > 0.5) with *Megamonas*, while IBA and IVA showed no consistent significant correlations with any genera.

### Functional predictions analysis

**Fig 5:**
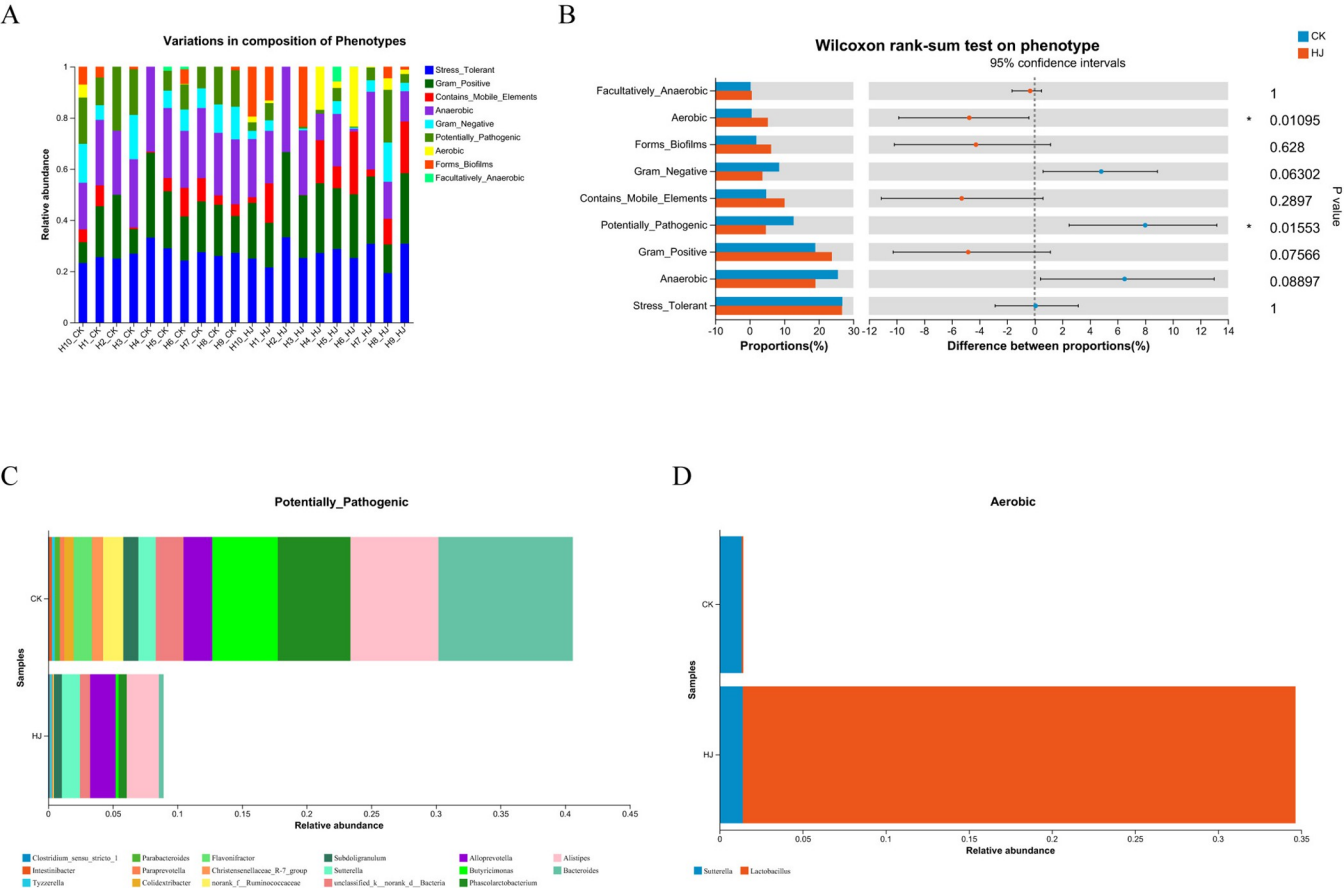
BugBase phenotype prediction of gut microbiota after *In vitro* simulated fermentation. (A) Variations in composition of phenotypes between the CK and HJ groups. (B) Wilcoxon rank-sum test on phenotype. The significance thresholds for P-values are * 0.01 < P ≤ 0.05, ** 0.001 < P ≤ 0.01, *** P ≤ 0.001. (C-D) Genera-phenotype contribution map of pathogenic and aerobic phenotypes.

BugBase phenotype prediction identifies high levels of phenotypes present in control and treated samples, enabling phenotype prediction. These phenotypes include characteristics such as gram-positive, gram-negative, biofilm-forming, pathogenic, containing mobile elements, utilizing oxygen (including aerobic, anaerobic, and facultatively anaerobic), and tolerance to oxidative stress. Additional contribution and difference analyses help elucidate phenotypic alterations in microorganisms across disease progression, treatment, or prognostic stages. The analysis from BugBase highlighted the distribution of microbial phenotypes in different samples (Fig 5A). Significant differences in the relative abundance of potentially pathogenic and aerobic phenotypes were detected by comparing phenotypic groups (Fig 5B). Further investigation into the gut flora’s contribution to these phenotypes revealed that in the CK group, the combined contributions of five genera (*Alloprevotella*, *Butyricimonas*, *Phascolarctobacterium*, *Alistipes*, and *Bacteroides*) to potentially pathogenic bacteria and the contribution of Lactobacillus to aerobic bacteria exceeded 0.9. In contrast, a significant reduction occurred in the HJ group (Fig 5C and 5D).

## Discussion

This research investigated the potential effects of *Polygonati Rhizoma* on the gut microbiota of pregnant women with poor dietary habits during *In vitro* simulated fermentation. *In vitro* simulated fermentation provides a controlled environment that allows for precise manipulation of key variables, including temperature, pH, and substrate composition [22]. This closely mimics the conditions present in the gastrointestinal tract, which is of great benefit to researchers. This methodology ensures the reproducibility of results, which facilitates consistent comparisons across studies and enables the rapid screening of microbial interactions and metabolic activities without the ethical implications associated with human or animal subjects [26]. Moreover, it permits a comprehensive examination of particular bacterial taxa and their metabolic products, thus facilitating a deeper comprehension of the functions performed by diverse microorganisms in the fermentation process. Additionally, researchers can assess the impact of diverse dietary elements, including fibers and prebiotics, on gut microbiota, thereby illustrating the value of this approach in elucidating the dietary influences on gut health [27]. It offers important perspectives on microbial diversity, community composition, metabolic processes, and functional forecasts.

Pan/Core genera analyses are frequently utilized in microbial biodiversity studies to evaluate the total genera richness and the number of core genera as the sample size increases. This aids in determining the adequacy of the sequencing sample size [28]. The observed trend of stabilization in the total number of genera with an increase in sample size does not appear to be statistically significant. This may be attributed to the relatively small sample size, which can limit the ability to observe a clear plateau in rarefaction curves. Furthermore, the variability in microbial composition among the samples may have contributed to this result. The core genera analysis is in accordance with established practices in microbial biodiversity studies. The concept of core genera, which indicates stable microbial communities, is supported by previous studies that have highlighted its importance for understanding the richness and stability of gut microbiota [28,29]. The effect of changes in the diversity of the gut microbiota induced by *Polygonati Rhizoma* on the health of pregnant women is of considerable importance. Alterations in gut microbiota diversity during pregnancy have been associated with several maternal health issues, such as GDM, preterm birth, and maternal immune function [30–32]. The Chao and Ace indices are commonly utilized metrics for estimating microbial richness, whereas the Sobs index reflects the observed richness in a given sample. The absence of notable discrepancies in the Chao and Ace indices between the CK (control) and HJ (Polygonati Rhizoma-treated) groups indicates that, on average, the potential diversity of microbial taxa is comparable between the two groups. Nevertheless, the notable discrepancy in the Sobs index suggests the potential for discernible variations in the actual microbial taxa present within the samples. Therefore, understanding how *Polygonati Rhizoma* could potentially affect the diversity of gut microbiota in pregnant women could shed light on its possible benefits in reducing pregnancy-related health risks. Further research in this area is essential to further understand the specific mechanisms of these effects and optimizing *Polygonati Rhizoma*’s therapeutic potential for pregnant women.

While *Escherichia-Shigella* was dominant in both groups, the observed increase in *Lactobacillus* and *Bifidobacterium* in the HJ group suggests a positive effect of *Polygonati Rhizoma* intervention on beneficial bacteria during pregnancy. The specific impact of *Polygonati Rhizoma* on *Lactobacillus* and *Bifidobacterium*, though not widely studied previously, can be supported by recent research that highlights the herb’s prebiotic potential and its capacity to selectively promote beneficial bacterial populations. It has been reported that increasing the abundance of *Lactobacillus* and *Bifidobacterium* was negatively correlated with serum and hepatic triglycerides (TG) and positively correlated with hepatic glycogen. Conversely, a decrease in the abundance of *Escherichia-Shigella* was positively correlated with serum and hepatic TG and negatively with hepatic glycogen [33]. This specificity highlights the necessity for more focused research into how *Polygonati Rhizoma* influences specific microbial populations.

The co-occurrence network analysis offers multiple advantages in microbiology. It aids in understanding the distribution of dominant genera across various samples, and also sheds light on their interactions and relationships with other microbial populations. This analytical approach allows for a deeper insight into the complex dynamics and ecological roles within microbial communities [34]. The co-occurrence network analysis results, which revealed unique and shared genera between the CK and HJ groups, provide valuable insights into the effects of Polygonati Rhizoma on gut microbiota in pregnant women with poor dietary habits. The differentiation of microbial genera between the CK and HJ groups underscores the influence of *Polygonati Rhizoma* on gut microbiota composition. For pregnant women, maintaining a balanced gut microbiota is critical for both maternal and fetal health [7]. The findings suggest that *Polygonati Rhizoma* may have a role in modulating gut microbiota, potentially counteracting the negative effects of poor dietary habits. The unique genera in each group may possess specific metabolic functions that contribute differently to health and disease states [35,36]. An understanding of these distinctions can inform the development of interventions based on Polygonati Rhizoma that promote the growth of beneficial microbes and suppress potentially harmful ones, thereby supporting maternal and fetal health. The RDA scatterplot and the Mantel-test network heatmap provided further insights into how samples are distributed and how microbial metabolites influence community structures within the samples [37,38]. The RDA scatterplot indicated that CO_2_ was more pronounced in the CK group, whereas PA was more noticeable in the HJ group. The Mantel-test network heatmap revealed significant correlations between IBA and IVA, and between H_2_S and NH_3_. The metabolites above are byproducts of microbial fermentation and protein degradation, respectively [36,39]. This indicates that specific metabolic pathways are actively engaged in response to *Polygonati Rhizoma* supplement. The elucidation of these correlations facilitates the comprehension of the metabolic characteristics shaped by the gut microbiota, which is of significance for the comprehension of the impact of *Polygonati Rhizoma* on metabolic processes in pregnant women with poor dietary habits.

The correlation heatmap plot displays the relationship between various genera and metabolites, showing the strength of correlation between different metabolites and various genera, and indicating whether these differences are statistically significant [40]. The results indicated that the genera *Parabacteroides*, *Lachnoclostridium*, and *Flavonifractor* showed strong positive correlations with the metabolites H_2_S and NH_3_, suggesting that these bacteria may play a role in the production or metabolism of these substances. H_2_S and NH_3_ are both compounds produced by the breakdown of organic matter, often by anaerobic bacteria in the gut [41,42]. The positive correlations found between these metabolites and the three bacterial genera clearly suggest that *Parabacteroide*s, *Lachnoclostridium*, and *Flavonifractor* may play a vital role in the generation or processing of H_2_S and NH_3_ in the gut microbiota. This is an important finding, as H_2_S and NH_3_ have significant physiological impacts, which can be either beneficial or harmful depending on their concentrations [41,42]. Thus, understanding which microbes contribute to the production of these metabolites can shed light on how *Polygonati Rhizoma* affects the gut health of pregnant women.

The BugBase approach enables researchers to advance beyond mere taxonomic identification of gut microbes to assess their potential roles and behaviors within the gut ecosystem. By classifying phenotypes such as gram-positive, gram-negative, biofilm-forming, and pathogenic characteristics, the study offers a nuanced understanding of how these microbial traits may relate to health conditions [43,44]. The identification of significant differences in the abundance of pathogenic and aerobic phenotypes between the control (CK) and treatment (HJ) groups provides insight into the potential health risks associated with specific microbial profiles. For example, the presence of pathogenic bacteria in the CK group may indicate an elevated risk of infection or inflammatory responses, underscoring the necessity for dietary interventions to mitigate these risks. Notably, in the CK group, five specific genera (*Alloprevotella*, *Butyricimonas*, *Phascolarctobacterium*, *Alistipes*, and *Bacteroides*) demonstrated a high combined contribution to potentially pathogenic bacteria, while Lactobacillus was significantly linked to aerobic bacteria. In contrast, the HJ group showed a marked reduction in these phenotypes. The ability to predict microbial phenotypes enables researchers to identify not just the presence of bacteria but also their functional implications with regard to health and disease. This predictive capacity enhances the understanding of how gut microbiota composition may influence the health of pregnant women, particularly those with poor dietary habits, thus guiding targeted interventions. The results of the BugBase analysis provide a basis for further investigation into the potential involvement of the gut microbiota in pregnancy-related health outcomes. By elucidating the phenotypic traits of gut microbes, future studies can explore the mechaccnisms by which dietary habits influence these traits and their subsequent effects on maternal and fetal health.

*In vitro* simulated fermentation serves as a valuable tool for studying the effects of various substances on the gut microbiota, particularly in contexts where ethical constraints hinder direct investigation in pregnant women. While *in vitro* simulated fermentation offers a number of advantages, it is not without limitations, which researchers must take into account. One of the primary limitations of *in vitro* simulated fermentation is the inability to fully replicate the complex and dynamic environment of the human gut. The interactions between microbiota and host, as well as the influence of immune responses on microbial behavior, are not fully captured *in vitro*. Since the study was conducted using *in vitro* simulated fermentation methods, the results might not fully capture the complexities of how *Polygonati Rhizoma* interacts with the gut microbiota in a living system. The study focused on pregnant women associated with poor dietary habits. Therefore, the results may not be generalizable to all pregnant women, especially those with different dietary patterns or underlying health conditions. This study does not mention the duration of the observation periods after administering *Polygonati Rhizoma*. Short-term analyses might not reflect long-term effects and dynamics in microbial communities, which could be crucial for understanding the overall impact on maternal and fetal health.

## Conclusion

This study explored the impact of *Polygonati Rhizoma* on the gut microbiota of pregnant women associated with poor dietary habits, using in vitro analyses. The research highlighted changes in microbial diversity, composition, metabolites, and functional predictions, providing novel perspective into the complex relationship between *Polygonati Rhizoma* and pregnant women with poor dietary habits. These findings emphasize the possibility of *Polygonati Rhizoma* as a prebiotic supplement, but short-term fermentation in vitro cannot fully replicate the *in vivo* process of *Polygonati Rhizoma*, and further *in vivo* studies are needed to obtain better confirmation.

## References

[1] Mousa A, Naqash A, Lim S. Macronutrient and Micronutrient Intake during Pregnancy: An Overview of Recent Evidence. Nutrients. 2019;11(2). Epub 20190220. doi: 10.3390/nu11020443 ; PubMed Central PMCID: PMC6413112. 30791647 PMC6413112

[2] Lotankar M, Mokkala K, Houttu N, Koivuniemi E, Sorensen N, Nielsen HB, et al. Distinct Diet-Microbiota-Metabolism Interactions in Overweight and Obese Pregnant Women: a Metagenomics Approach. Microbiol Spectr. 2022;10(2):e0089321. Epub 20220328. doi: 10.1128/spectrum.00893-21 ; PubMed Central PMCID: PMC9045358. 35343768 PMC9045358

[3] Beam A, Clinger E, Hao L. Effect of Diet and Dietary Components on the Composition of the Gut Microbiota. Nutrients. 2021;13(8). Epub 20210815. doi: 10.3390/nu13082795 ; PubMed Central PMCID: PMC8398149. 34444955 PMC8398149

[4] Yu J, Zhang B, Miao T, Hu H, Sun Y. Dietary Nutrition and Gut Microbiota Composition in Patients With Hypertensive Disorders of Pregnancy. Front Nutr. 2022;9:862892. Epub 20220406. doi: 10.3389/fnut.2022.862892 ; PubMed Central PMCID: PMC9019690. 35464021 PMC9019690

[5] Ruebel ML, Gilley SP, Sims CR, Zhong Y, Turner D, Chintapalli SV, et al. Associations between Maternal Diet, Body Composition and Gut Microbial Ecology in Pregnancy. Nutrients. 2021;13(9). Epub 20210921. doi: 10.3390/nu13093295 ; PubMed Central PMCID: PMC8468685. 34579172 PMC8468685

[6] Tang M, Weaver NE, Frank DN, Ir D, Robertson CE, Kemp JF, et al. Longitudinal Reduction in Diversity of Maternal Gut Microbiota During Pregnancy Is Observed in Multiple Low-Resource Settings: Results From the Women First Trial. Front Microbiol. 2022;13:823757. Epub 20220801. doi: 10.3389/fmicb.2022.823757 ; PubMed Central PMCID: PMC9376441. 35979501 PMC9376441

[7] Sajdel-Sulkowska EM. The Impact of Maternal Gut Microbiota during Pregnancy on Fetal Gut-Brain Axis Development and Life-Long Health Outcomes. Microorganisms. 2023;11(9). Epub 20230831. doi: 10.3390/microorganisms11092199 ; PubMed Central PMCID: PMC10538154. 37764043 PMC10538154

[8] Aizawa S, Uebanso T, Shimohata T, Mawatari K, Takahashi A. Effects of the loss of maternal gut microbiota before pregnancy on gut microbiota, food allergy susceptibility, and epigenetic modification on subsequent generations. Biosci Microbiota Food Health. 2023;42(3):203–12. Epub 20230405. doi: 10.12938/bmfh.2022-093 ; PubMed Central PMCID: PMC10315195. 37404565 PMC10315195

[9] Bankole T, Winn H, Li Y. Dietary Impacts on Gestational Diabetes: Connection between Gut Microbiome and Epigenetic Mechanisms. Nutrients. 2022;14(24). Epub 20221210. doi: 10.3390/nu14245269 ; PubMed Central PMCID: PMC9786016. 36558427 PMC9786016

[10] Li P, Wang H, Guo L, Gou X, Chen G, Lin D, et al. Association between gut microbiota and preeclampsia-eclampsia: a two-sample Mendelian randomization study. BMC Med. 2022;20(1):443. Epub 20221115. doi: 10.1186/s12916-022-02657-x ; PubMed Central PMCID: PMC9667679. 36380372 PMC9667679

[11] Cirulli F, De Simone R, Musillo C, Ajmone-Cat MA, Berry A. Inflammatory Signatures of Maternal Obesity as Risk Factors for Neurodevelopmental Disorders: Role of Maternal Microbiota and Nutritional Intervention Strategies. Nutrients. 2022;14(15). Epub 20220730. doi: 10.3390/nu14153150 ; PubMed Central PMCID: PMC9370669. 35956326 PMC9370669

[12] Cheng R, Zhang Y, Yang Y, Ren L, Li J, Wang Y, et al. Maternal gestational Bifidobacterium bifidum TMC3115 treatment shapes construction of offspring gut microbiota and development of immune system and induces immune tolerance to food allergen. Front Cell Infect Microbiol. 2022;12:1045109. Epub 20221114. doi: 10.3389/fcimb.2022.1045109 ; PubMed Central PMCID: PMC9701730. 36452299 PMC9701730

[13] Lu X, Shi Z, Jiang L, Zhang S. Maternal gut microbiota in the health of mothers and offspring: from the perspective of immunology. Front Immunol. 2024;15:1362784. Epub 20240313. doi: 10.3389/fimmu.2024.1362784 ; PubMed Central PMCID: PMC10965710. 38545107 PMC10965710

[14] Nie P, Li Z, Wang Y, Zhang Y, Zhao M, Luo J, et al. Gut microbiome interventions in human health and diseases. Med Res Rev. 2019;39(6):2286–313. Epub 20190417. doi: 10.1002/med.21584 . 30994937

[15] Koren G, Klinger G, Ohlsson A. Fetal pharmacotherapy. Drugs. 2002;62(5):757–73. doi: 10.2165/00003495-200262050-00004 . 11929330

[16] Luo M, Hu Z, Zhong Z, Liu L, Lin C, He Q. Chemical Structures and Pharmacological Properties of Typical Bioflavonoids in Polygonati Rhizoma (PGR). J Environ Public Health. 2022;2022:4649614. Epub 20220920. doi: 10.1155/2022/4649614 ; PubMed Central PMCID: PMC9788903. 36570783 PMC9788903

[17] Shi Y, Yang TG, Yang MS, Yu M, Zhang XF. [Polygonati Rhizoma: a crop with potential of being consumed as food and medicine]. Zhongguo Zhong Yao Za Zhi. 2022;47(4):1132–5. doi: 10.19540/j.cnki.cjcmm.20211105.101 . 35285215

[18] Benassi E, Fan H, Sun Q, Dukenbayev K, Wang Q, Shaimoldina A, et al. Generation of particle assemblies mimicking enzymatic activity by processing of herbal food: the case of rhizoma polygonati and other natural ingredients in traditional Chinese medicine. Nanoscale Adv. 2021;3(8):2222–35. Epub 20210113. doi: 10.1039/d0na00958j ; PubMed Central PMCID: PMC9417895. 36133773 PMC9417895

[19] Su LL, Li X, Guo ZJ, Xiao XY, Chen P, Zhang JB, et al. Effects of different steaming times on the composition, structure and immune activity of Polygonatum Polysaccharide. J Ethnopharmacol. 2023;310:116351. Epub 20230311. doi: 10.1016/j.jep.2023.116351 . 36914038

[20] Wang S, He F, Wu H, Xiang F, Zheng H, Wu W, et al. Health-Promoting Activities and Associated Mechanisms of Polygonati Rhizoma Polysaccharides. Molecules. 2023;28(3). Epub 20230131. doi: 10.3390/molecules28031350 ; PubMed Central PMCID: PMC9919897. 36771015 PMC9919897

[21] Leppala J, Lagstrom H, Kaljonen A, Laitinen K. Construction and evaluation of a self-contained index for assessment of diet quality. Scand J Public Health. 2010;38(8):794–802. Epub 20100916. doi: 10.1177/1403494810382476 . 20846997

[22] Pi XE, Fu H, Yang XX, Yu ZC, Teng WL, Zhang Y, et al. Bacterial, short-chain fatty acid and gas profiles of partially hydrolyzed guar gum in vitro fermentation by human fecal microbiota. Food Chem. 2024;430:137006. Epub 20230727. doi: 10.1016/j.foodchem.2023.137006 . 37541036

[23] Chen J, Pi X, Liu W, Ding Q, Wang X, Jia W, et al. Age-related changes of microbiota in midlife associated with reduced saccharolytic potential: an in vitro study. BMC Microbiol. 2021;21(1):47. Epub 20210215. doi: 10.1186/s12866-021-02103-7 ; PubMed Central PMCID: PMC7885556. 33588748 PMC7885556

[24] Pi X, Hua H, Wu Q, Wang X, Wang X, Li J. Effects of Different Feeding Methods on the Structure, Metabolism, and Gas Production of Infant and Toddler Intestinal Flora and Their Mechanisms. Nutrients. 2022;14(8). Epub 20220409. doi: 10.3390/nu14081568 ; PubMed Central PMCID: PMC9027170. 35458130 PMC9027170

[25] Ye X, Pi X, Zheng W, Cen Y, Ni J, Xu L, et al. The Methanol Extract of Polygonatum odoratum Ameliorates Colitis by Improving Intestinal Short-Chain Fatty Acids and Gas Production to Regulate Microbiota Dysbiosis in Mice. Front Nutr. 2022;9:899421. Epub 20220512. doi: 10.3389/fnut.2022.899421 ; PubMed Central PMCID: PMC9133717. 35634366 PMC9133717

[26] Li C, Zhang X. Current in Vitro and Animal Models for Understanding Foods: Human Gut-Microbiota Interactions. J Agric Food Chem. 2022;70(40):12733–45. Epub 20220927. doi: 10.1021/acs.jafc.2c04238 . 36166347

[27] Perez-Burillo S, Molino S, Navajas-Porras B, Valverde-Moya AJ, Hinojosa-Nogueira D, Lopez-Maldonado A, et al. An in vitro batch fermentation protocol for studying the contribution of food to gut microbiota composition and functionality. Nat Protoc. 2021;16(7):3186–209. Epub 20210604. doi: 10.1038/s41596-021-00537-x . 34089022

[28] Wu Y, Zaiden N, Cao B. The Core- and Pan-Genomic Analyses of the Genus Comamonas: From Environmental Adaptation to Potential Virulence. Front Microbiol. 2018;9:3096. Epub 20181212. doi: 10.3389/fmicb.2018.03096 ; PubMed Central PMCID: PMC6299040. 30619175 PMC6299040

[29] Joglekar P, Conlan S, Lee-Lin SQ, Deming C, Kashaf SS, Program NCS, et al. Integrated genomic and functional analyses of human skin-associated Staphylococcus reveals extensive inter- and intra-species diversity. bioRxiv. 2023. Epub 20230623. doi: 10.1101/2023.06.22.546190 ; PubMed Central PMCID: PMC10370188. 37956283 PMC10666031

[30] Lv Y, Yan Z, Zhao X, Gang X, He G, Sun L, et al. The effects of gut microbiota on metabolic outcomes in pregnant women and their offspring. Food Funct. 2018;9(9):4537–47. doi: 10.1039/c8fo00601f . 30101246

[31] Gershuni V, Li Y, Elovitz M, Li H, Wu GD, Compher CW. Maternal gut microbiota reflecting poor diet quality is associated with spontaneous preterm birth in a prospective cohort study. Am J Clin Nutr. 2021;113(3):602–11. doi: 10.1093/ajcn/nqaa361 ; PubMed Central PMCID: PMC7948858. 33515003 PMC7948858

[32] Long ES, Penalver Bernabe B, Xia K, Azcarate-Peril MA, Carroll IM, Rackers HS, et al. The microbiota-gut-brain axis and perceived stress in the perinatal period. Arch Womens Ment Health. 2023;26(2):227–34. Epub 20230310. doi: 10.1007/s00737-023-01300-9 ; PubMed Central PMCID: PMC10063483. 36897389 PMC10063483

[33] Tang Z, Luo T, Huang P, Luo M, Zhu J, Wang X, et al. Nuciferine administration in C57BL/6J mice with gestational diabetes mellitus induced by a high-fat diet: the improvement of glycolipid disorders and intestinal dysbacteriosis. Food Funct. 2021;12(22):11174–89. Epub 20211115. doi: 10.1039/d1fo02714j . 34636388

[34] Cui X, He H, Zhu F, Liu X, Ma Y, Xie W, et al. Community structure and co-occurrence network analysis of bacteria and fungi in wheat fields vs fruit orchards. Arch Microbiol. 2022;204(8):453. Epub 20220704. doi: 10.1007/s00203-022-03074-7 . 35786781

[35] Conteville LC, Vicente ACP. A plasmid network from the gut microbiome of semi-isolated human groups reveals unique and shared metabolic and virulence traits. Sci Rep. 2022;12(1):12102. Epub 20220715. doi: 10.1038/s41598-022-16392-z ; PubMed Central PMCID: PMC9287393. 35840779 PMC9287393

[36] Nicholson JK, Holmes E, Kinross J, Burcelin R, Gibson G, Jia W, et al. Host-gut microbiota metabolic interactions. Science. 2012;336(6086):1262–7. Epub 20120606. doi: 10.1126/science.1223813 . 22674330

[37] Zhang J, He X, Zhang H, Liao Y, Wang Q, Li L, et al. Factors Driving Microbial Community Dynamics and Potential Health Effects of Bacterial Pathogen on Landscape Lakes with Reclaimed Water Replenishment in Beijing, PR China. Int J Environ Res Public Health. 2022;19(9). Epub 20220422. doi: 10.3390/ijerph19095127 ; PubMed Central PMCID: PMC9106022. 35564521 PMC9106022

[38] Meng Q, Liu S, Guo Y, Hu Y, Yu Z, Bello A, et al. The co-occurrence network patterns and keystone species of microbial communities in cattle manure-corn straw composting. Environ Sci Pollut Res Int. 2023;30(8):20265–76. Epub 20221017. doi: 10.1007/s11356-022-23599-0 . 36251182

[39] Verbeke KA, Boobis AR, Chiodini A, Edwards CA, Franck A, Kleerebezem M, et al. Towards microbial fermentation metabolites as markers for health benefits of prebiotics. Nutr Res Rev. 2015;28(1):42–66. doi: 10.1017/S0954422415000037 ; PubMed Central PMCID: PMC4501371. 26156216 PMC4501371

[40] Narihiro T, Nobu MK, Bocher BTW, Mei R, Liu WT. Co-occurrence network analysis reveals thermodynamics-driven microbial interactions in methanogenic bioreactors. Environ Microbiol Rep. 2018;10(6):673–85. Epub 20180926. doi: 10.1111/1758-2229.12689 . 30136425

[41] Kalantar-Zadeh K, Berean KJ, Burgell RE, Muir JG, Gibson PR. Intestinal gases: influence on gut disorders and the role of dietary manipulations. Nat Rev Gastroenterol Hepatol. 2019;16(12):733–47. Epub 20190913. doi: 10.1038/s41575-019-0193-z . 31520080

[42] Hopper CP, De La Cruz LK, Lyles KV, Wareham LK, Gilbert JA, Eichenbaum Z, et al. Role of Carbon Monoxide in Host-Gut Microbiome Communication. Chem Rev. 2020;120(24):13273–311. Epub 20201022. doi: 10.1021/acs.chemrev.0c00586 . 33089988

[43] Wu H, Wang Y, Dong L, Hu H, Meng L, Liu H, et al. Microbial Characteristics and Safety of Dairy Manure ComPosting for Reuse as Dairy Bedding. Biology (Basel). 2020;10(1). Epub 20201228. doi: 10.3390/biology10010013 ; PubMed Central PMCID: PMC7824547. 33379325 PMC7824547

[44] Gong C, Yang L, Liu K, Shen S, Zhang Q, Li H, et al. Effects of Antibiotic Treatment and Probiotics on the Gut Microbiome of 40 Infants Delivered Before Term by Cesarean Section Analysed by Using 16S rRNA Quantitative Polymerase Chain Reaction Sequencing. Med Sci Monit. 2021;27:e928467. Epub 20210205. doi: 10.12659/MSM.928467 ; PubMed Central PMCID: PMC7871509. 33542172 PMC7871509

