# Correction: Characteristics of the effects of *Polygonati Rhizoma* on gut microbiota and metabolites in vitro associated with poor dietary habits in pregnant women

**DOI:** 10.1371/journal.pone.0332056

**Published:** 2025-09-09

**Authors:** Zhiwei Xu, Jiabin Li, Lue Hong, Yangli Zhang, Chunyu Wang, Hailong Yang, Lisha Zhao, Ping Qiu, Zhi Du, Hui Wang

The first three authors, Zhiwei Xu, Jiabin Li, and Lue Hong, should be noted as contributing equally as first authors on this work.
